# Influence of atorvastatin on metabolic pattern of rats with pulmonary hypertension

**DOI:** 10.18632/aging.202898

**Published:** 2021-04-22

**Authors:** Li Luo, Jianmin Wu, Taijie Lin, Guili Lian, Huajun Wang, Gufeng Gao, Liangdi Xie

**Affiliations:** 1 Department of Geriatrics, The First Affiliated Hospital of Fujian Medical University, Fuzhou, China; 2 Fujian Hypertension Research Institute, The First Affiliated Hospital of Fujian Medical University, Fuzhou, China; 3 Fujian Medical University, Fuzhou, China

**Keywords:** metabonomics, pulmonary arterial hypertension, monocrotaline, Warburg effect, fatty acid β oxidation

## Abstract

Background: Metabonomics has been widely used to analyze the initiation, progress, and development of diseases. However, application of metabonomics to explore the mechanism of pulmonary arterial hypertension (PAH) are poorly reported. This study aimed to investigate the influence of atorvastatin (Ato) on metabolic pattern of rats with pulmonary hypertension.

Methods: PAH animal model was established using monocrotaline (MCT). The mean pulmonary artery pressure (mPAP) and right ventricular hypertrophy index (RVHI) were measured. The microstructure of pulmonary arterioles was observed by HE staining. Nuclear magnetic resonance was used to detect and analyze the serum metabolites. The levels of glycogen synthase kinase-3β (GSK-3β), hexokinase 2 (HK-2), sterol regulatory element-binding protein 1c (SREBP-1c), and carnitine palmitoyltransferase I (CPT-1) in the lung tissues were measured.

Results: Ato significantly improved lung function by decreasing mPAP, RVHI, wall thickness, and wall area. Differences in metabolic patterns were observed among normal, PAH, and Ato group. The levels of GSK-3β and SREBP-1c were decreased, but HK-2 and CPT-1 were increased in the group PAH. Ato treatment markedly reversed the influence of MCT.

Conclusion: Ato significantly improved the pulmonary vascular remodeling and pulmonary hypertension of PAH rats due to its inhibition on Warburg effect and fatty acid β oxidation.

## INTRODUCTION

Pulmonary arterial hypertension (PAH) is a group of clinical pathophysiological syndrome, caused by various reasons. The mean pulmonary arterial pressure (mPAP) measured by right heart catheterization is more than 25 mmHg [1, 2]. The pathogenesis of PAH is very complex and has not been fully elucidated. It is believed that the main pathogenesis of PAH is the abnormal proliferation of smooth muscle cells, damage of fibroblasts and endothelial cells. The dysfunction of cells results in pulmonary vascular remodeling, and further leads to pulmonary artery lumen obstruction. Pulmonary vascular remodeling is believed to be the main reason for the continuous increase of pulmonary artery pressure [3–5]. However, the specific mechanisms during the initiation and progress of PAH have not been fully understood.

Statins, known as 3-hydroxy-3-methylglutaryl coenzyme A (HMG-CoA) reductase inhibitors, are widely used in the treatment of patients with elevated blood cholesterol, including atorvastatin (Ato), simvastatin, pravastatin, and so on [6]. Previous studies have found that atorvastatin could significantly reduce the pulmonary artery pressure of PAH rats induced by MCT, and can reverse the vascular remodeling during pulmonary hypertension [7]. Meanwhile, pulmonary vascular remodeling has already occurred before the change of pressure, but its mechanism has not been well elucidated. In addition, Ato can inhibit neointimal formation, smooth muscle cell proliferation and migration [8–10]. However, the mechanism of it improving vascular remodeling remains unclear.

In recent years, metabonomics has been widely used to develop biomarkers for several diseases [11]. Studies have shown that it is more sensitive than traditional diagnostic methods and can improve the diagnosis rate of diseases [12]. Metabonomics can be used not only for clinical diagnosis, but also for evaluating the clinical course of disease, the prognosis of patients, and the efficacy of surgery or drugs [13, 14]. The research on the metabolic model of diseases can help people understand the pathological process and deepen the understanding of some diseases. At present, there are few studies on the application of metabonomics to PAH. Increased biosynthesis of fatty acids was observed in the PAH birds measured using metabonomics profiling [15]. The differences of biologic mechanisms between exercise pulmonary hypertension and PAH were analyzed using metabonomics. They found that PAH group presented perturbations in several pathways such as collagen deposition, fatty acid, and glycolysis [16]. In addition, metabolomic profiles were conducted to differentiate PAH and idiopathic PAH [17]. However, the application of metabolomics in investigating how Ato improve PAH has not been reported.

Monocrotaline (MCT) is an alkaloid of phytotoxin, which is often used to establish animal models of pulmonary hypertension [18, 19]. In this study, MCT (60mg/kg) was used to establish PAH animal model. The aim of this study was to observe the changes of pulmonary artery remodeling and pulmonary arterial pressure in rats with pulmonary hypertension at different time points after atorvastatin treatment. Serum metabolic patterns were detected simultaneously to find the characteristic metabolites, and the relationship between pulmonary artery remodeling and metabolic model changes was analyzed. glycogen synthase kinase-3β (GSK-3β), hexokinase 2 (HK-2), sterol regulatory element-binding protein 1c (SREBP-1c), and carnitine palmitoyltransferase I (CPT-1) were detected to explore the possibility and mechanism of Ato in the treatment of PAH. This study might provide novel thought for the diagnose, prevention, and treatment of PAH.

## RESULTS

### Ato improved the lung function of PAH rats

**Figure 1:**
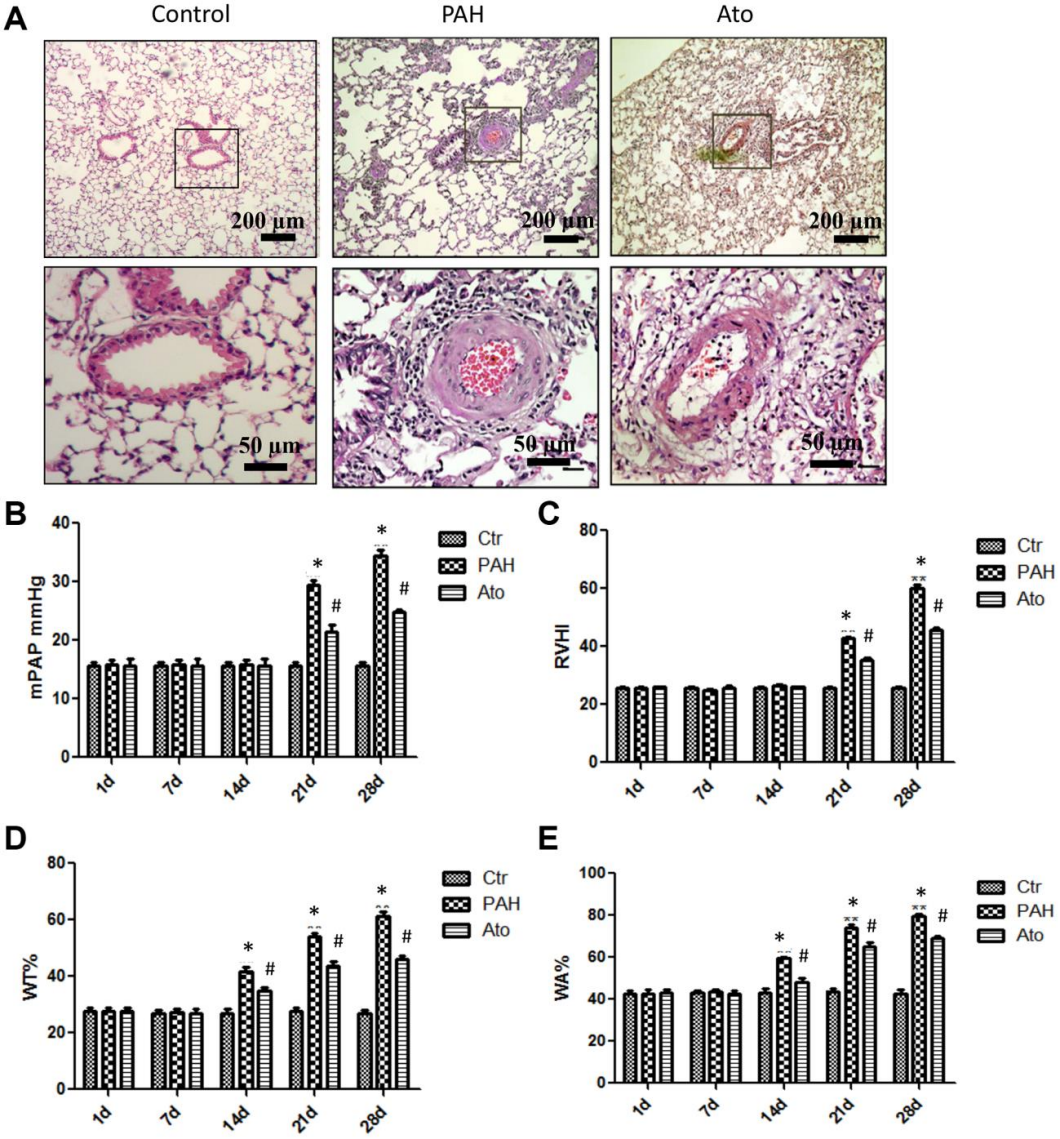
**Ato improved the lung function of PAH rats.** (**A**) Pathological changes of lung tissues after treatment with MCT and Ato; (**B**) mPAP of rats in different groups was measured at different time points; (**C**) RVHI of rats in different groups was measured at different time points; (**D**) WT was measured at different time points after treatment with MCT and Ato; (**E**) WA was measured at different time points after treatment with MCT and Ato. (*P<0.05, compared with control group, #P<0.05, compared with group PAH). Ten rats were used in each group.

In order to investigate the influence of Ato on PAH, PAH animal model was established. In the group PAH, remarkable alveoli distention, inflammatory cell infiltration, increased vessel walls, and structural disruption of the lung tissue were found indicating successful establishment of PAH model (Figure 1A). However, in the group Ato, decreased inflammatory cell infiltration and vessel walls, and relatively complete alveolar structure were observed (Figure 1A). To further investigate the effect of Ato on PAH, mPAP, RVHI, WT, and WA were measured. No significant differences were observed in terms of mPAP and RVHI after 1 day, 1 week, and 2 weeks between different groups (Figure 1B, 1C). However, after 3 and 4 weeks, the levels of mPAP and RVHI were increased significantly in group PAH compared with control, but Ato treatment could remarkably reversed these trends (Figure 1B, 1C). Similar findings were observed about changes of WT and WA. In the group PAH, the levels of WT and WA were promoted after 2, 3, and 4 weeks, but Ato remarkably decreased the values of them (Figure 1D, 1E). These results indicated that pulmonary artery remodeling appeared before the increase of pulmonary artery pressure, and Ato could improve pulmonary artery remodeling and reduce pulmonary artery pressure.

### PLS-DA analysis was used to investigate the influence of Ato on the metabonomics of PAH rats

**Figure 2:**
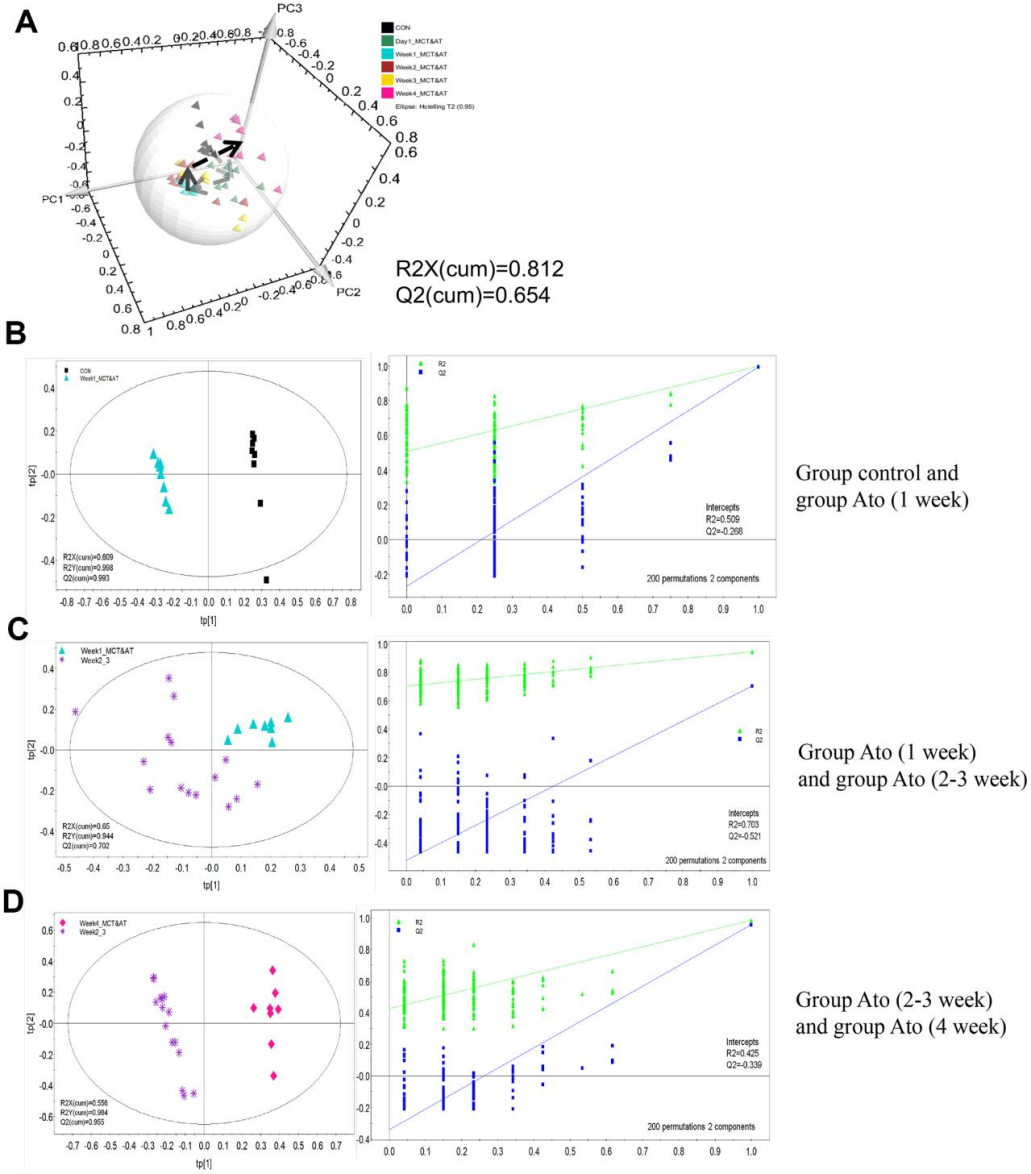
**PLS-DA analysis was used to investigate the influence of Ato on the metabonomics of PAH rats.** (**A**) The serum metabolic patterns of rats in different groups can be distinguished; (**B**) The metabonomics differences between group control and Ato (1 week) could be distinguished; (**C**) The metabonomics differences between group Ato (1 week) and Ato (2-3 week) could be distinguished; (**D**) The metabonomics differences between group Ato (2-3 week) and Ato (4 week) could be distinguished.

In order to investigate the influence of Ato on the metabonomics of PAH rats, PLS-DA method was conducted. The metabolic patterns of rats’ serum in different groups can be distinguished (Figure 2A). PLS-DA method was used to analyze metabonomics difference between different groups. The metabonomics differences between group control and Ato (1 week), group Ato (1 week) and Ato (2-3 week), group Ato (2-3 week) and Ato (4 week) could be distinguished (Figure 2B–2D). Meanwhile, the analysis results were verified by response sequencing (Right panels of Figure 2B–2D) indicating that PLS-DA method was effective.

### OPLS-DA analysis was used to find differential metabolites between differnent groups

**Figure 3:**
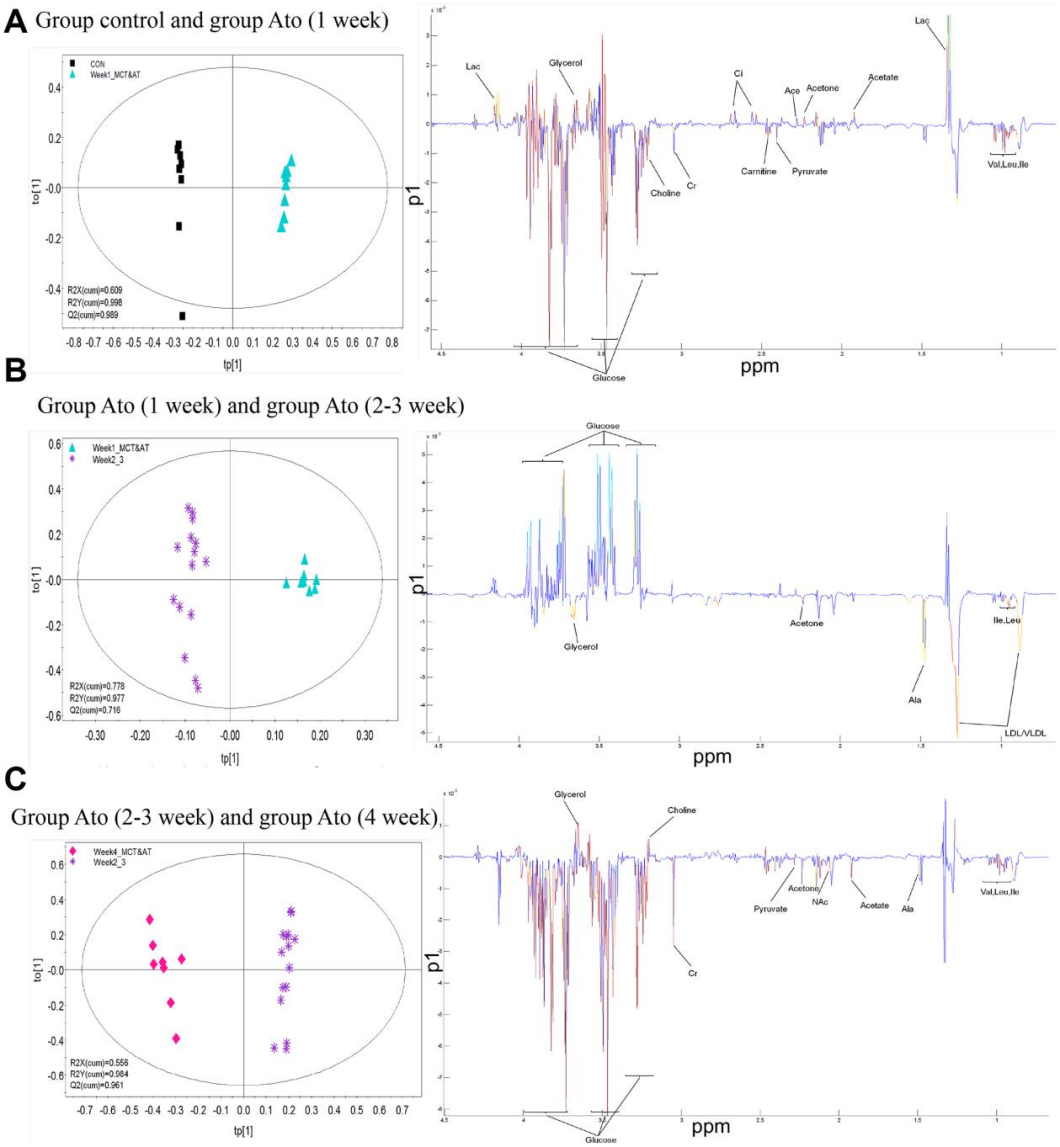
**OPLS-DA analysis was used to find differential metabolites between different groups.** (**A**) Differential metabolites were investigated between group control and Ato (1 week); (**B**) Differential metabolites were investigated between group Ato (1 week) and Ato (2-3 week); (**C**) Differential metabolites were investigated between group Ato (2-3 week) and Ato (4 week).

The serum metabolic patterns of group control and Ato (1 week) in the first predictive principal component (tp1) of was easily distinguished using OPLS-DA method (Left panel of Figure 3A). Variable importance in projection (VIP) and correlation coefficients (r) were performed to analyze tp1. The variables with VIP>1 and |r| exceeding the threshold were screened out (Right panel of Figure 3A), and differential metabolites were listed in the Table 1. Same method was applied to analyze differential metabolites between group Ato (1 week) and Ato (2-3 week), group Ato (2-3 week) and Ato (4 week). The differential metabolites were listed in the Tables 2, 3.

**Table 1:** Differential metabolites between week 1 and control.

**Chemical shift**	**Metabolites**	**|r|**	**VIP**	**Week 1 vs control**
0.995	Ile	0.9104	2.5786	↓
0.962	Leu	0.8279	1.8496	↓
1.049	Val	0.8404	2.0079	↓
1.336	Lac	0.5143	4.8674	↑
2.23	Acetone	0.7498	1.2493	↑
2.28	Ace	0.6531	1.0639	↑
2.38	Pyruvate	0.5546	1.0689	↑
2.445	Carnitine	0.6843	1.3674	↑
2.558	Ci	0.8637	1.7012	↑
3.04	Cr	0.4653	1.9865	↓
3.21	Choline	0.8358	1.7212	↓
3.25	Glucose	0.7718	3.3399	↓
3.67	Glycerol	0.8803	2.1916	↑

**Table 2:** Differential metabolites between 2-3 week and week 1.

**Chemical shift**	**Metabolites**	**|r|**	**VIP**	**Week 3 vs week 1**
0.894	LDL/VLDL	0.4897	3.1081	↑
0.983	Ile	0.5212	1.4465	↑
0.962	Leu	0.4431	1.6786	↑
2.23	Acetone	0.5387	1.4087	↑
3.25	Glucose	0.4604	4.8568	↓
3.67	Glycerol	0.4182	2.2569	↑

**Table 3:** Differential metabolites between week 4 and 2-3 week.

**Chemical shift**	**Metabolites**	**|r|**	**VIP**	**Week 4 vs week 3**
0.995	Ile	0.6073	1.4734	↑
0.962	Leu	0.75	1.3312	↑
1.04	Val	0.6463	1.1487	↑
1.48	Ala	0.5124	1.4160	↑
1.92	Acetate	0.7553	2.0562	↑
2.04	NAc	0.4873	1.4119	↑
2.23	Acetone	0.5525	1.3427	↑
2.28	Pyruvate	0.6875	1.2207	↑
3.04	Cr	0.6008	3.6761	↑
3.20	Choline	0.8041	1.9852	↑
3.25	Glucose	0.6353	3.0678	↓
3.67	Glycerol	0.6835	2.2646	↓

### Influence of Ato on the mRNA expression of GSK-3β, HK-2, SREBP-1c, and CPT-1 in the lung tissues of rats

**Figure 4:**
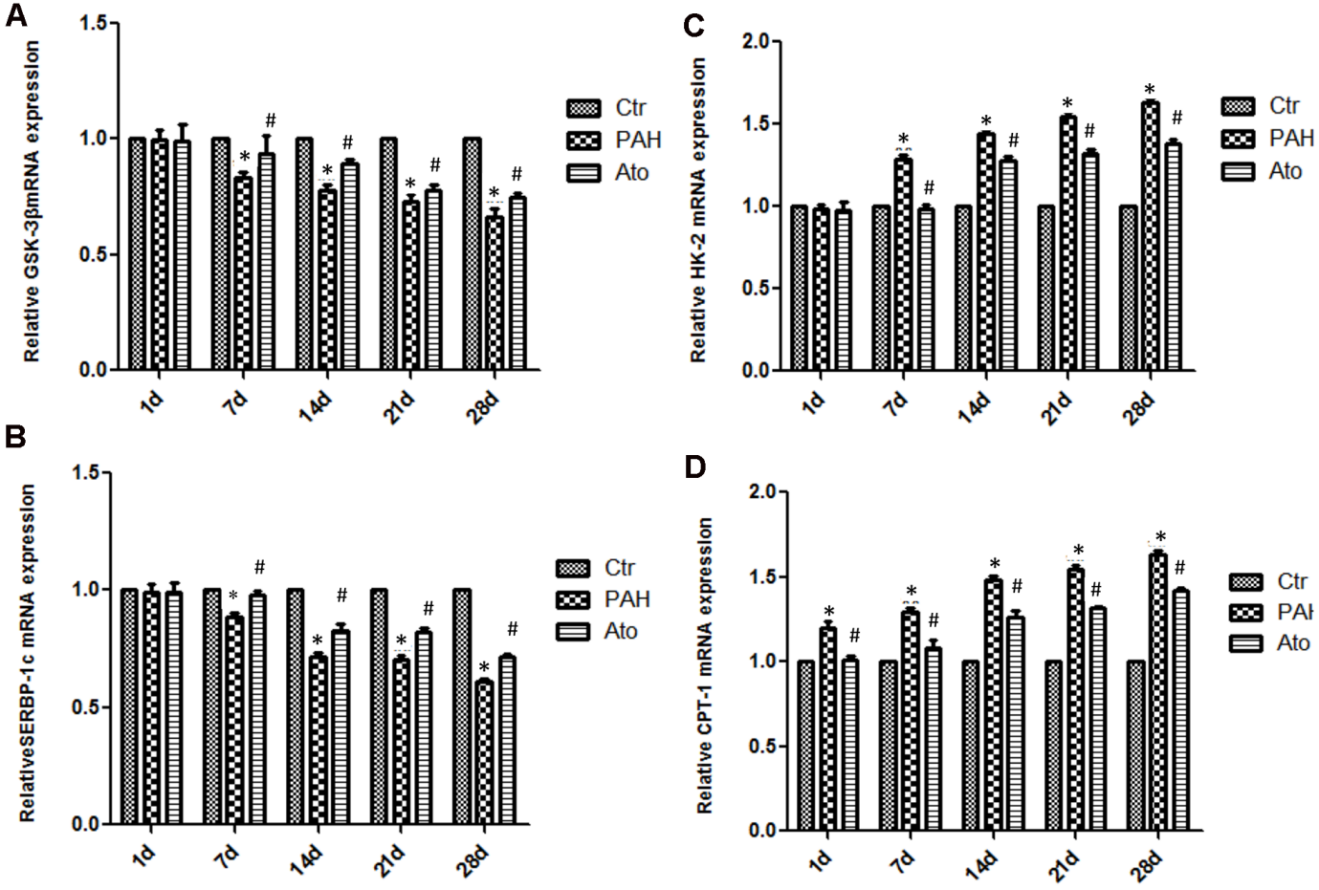
**Influence of Ato on the mRNA expression of GSK-3β, HK-2, SREBP-1c, and CPT-1 in the lung tissues.** (**A**) The mRNA level of GSK-3β was detected after treatment with MCT and Ato; (**B**) The mRNA level of HK-2 was detected after treatment with MCT and Ato; (**C**) The mRNA level of SREBP-1c was detected after treatment with MCT and Ato; (**D**) The mRNA level of CPT-1 was detected after treatment with MCT and Ato. (*P<0.05, compared with control group, #P<0.05, compared with group PAH).

The mRNA levels of GSK-3β and SERBP-1c in group PAH were significantly decreased after 1, 2, 3, and 4 weeks (Figure 4A, 4B). However, after treatment with Ato, the decreased trend of GSK-3β and SERBP-1c was reversed markedly. On the contrary, the mRNA levels of HK-2 and CPT -1 were remarkably promoted in the group PAH, but the influence of PAH was weakened significantly by Ato (Figure 4C, 4D).

### The protein expression changes of GSK-3β, HK-2, SREBP-1c, and CPT-1 in the group PAH

**Figure 5:**
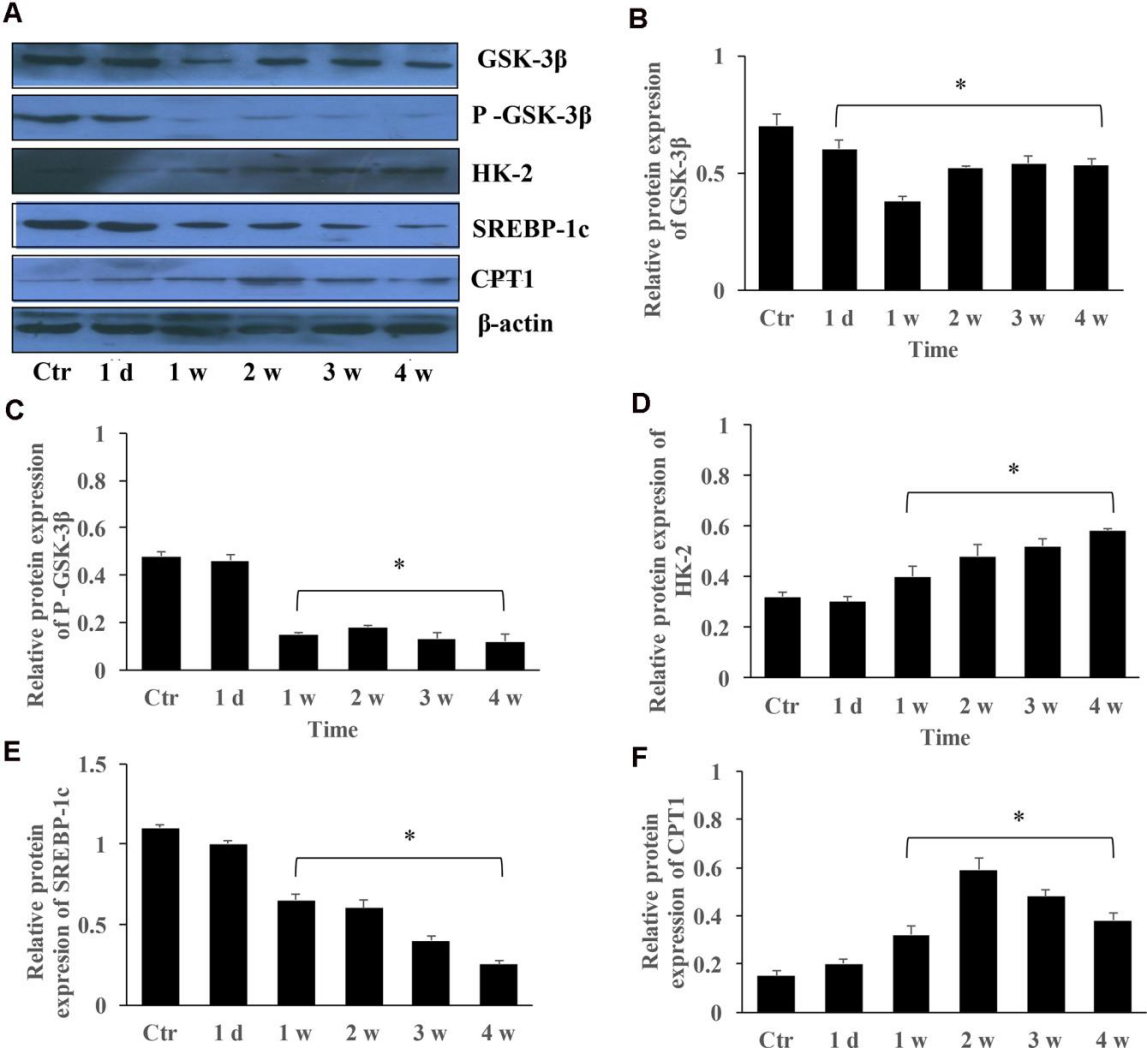
**The protein expression changes of GSK-3β, HK-2, SREBP-1c, and CPT-1 in the group PAH.** (**A**) The protein levels in the lung tissues of PAH rats was measured using western blotting; (**B**) The protein expression change of GSK-3β was quantified; (**C**) The protein expression of p-GSK-3β was quantified; (**D**) The protein expression of HK-2 in the lung tissues was quantified; (**E**) The protein expression of SREBP-1c was quantified; (**F**) The protein expression of CPT-1 was quantified. (*P<0.05, compared with control group).

The protein expression of GSK-3β, HK-2, SERBP-1c, and CPT-1 in the group PAH at different time points were measured. The levels of GSK-3β and p-GSK-3β were markedly inhibited in the group PAH after 1, 2, 3, and 4 weeks (Figure 5A–5C). However, the protein expression of HK-2 and CPT-1 were gradually promoted (Figure 5D, 5F). Meanwhile, the level of SERBP-1c was suppressed in the MCT induced PAH animal model (Figure 5E).

### Influence of Ato on the protein levels of GSK-3β, HK-2, SERBP-1c, and CPT-1 in the lung tissues

**Figure 6:**
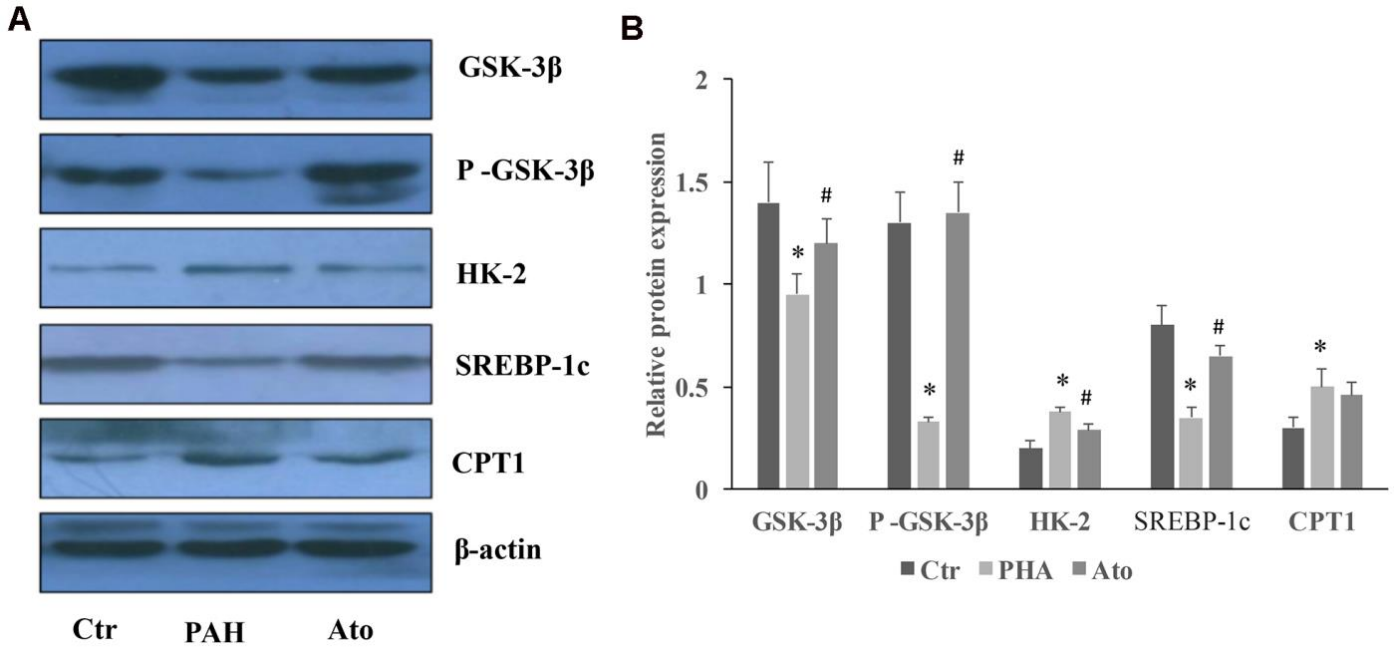
**Influence of Ato on the protein levels of GSK-3β, HK-2, SERBP-1c, and CPT-1 in the lung tissues.** (**A**) The protein levels in the lung tissues of Ato treated rats was measured using western blotting; (**B**) The protein expression was quantified.

The levels of GSK-3β, p-GSK-3β, and SERBP-1c were significantly suppressed by MCT in the group PAH, but the concentrations of HK-2 and CPT-1 were promoted (Figure 6A, 6B). However, Ato could remarkably reversed the influence of MCT by increasing GSK-3β, p-GSK-3β, and SERBP-1c, and decreasing HK-2 and CPT-1 (Figure 6A, 6B).

## DISCUSSION

The predisposing factors of PAH include environmental factors, genetic markers and acquired factors [20]. The main pathological changes of PAH are pulmonary vasospasm, intimal hyperplasia and remodeling, and the formation of microthrombotic lesions [4, 21]. In the early stage of PAH, the pathological changes are myogenic and increased number of pulmonary arterioles, obvious thickening of muscular pulmonary artery middle layer, extensive contraction and hyperplasia of arterial intimal cells. In the late stage of PAH, the main pathological changes are intimal fibrosis, hyperplasia of small arteries, obstruction of lumen, muscle pulmonary artery muscularization and occlusion, decrease of pulmonary vascular bed, formation of plexiform lesions, and necrotizing arteritis [22, 23].

In the present study, PAH animal model was established using MCT, and found that pulmonary vascular remodeling occurred before pressure rise. After 2 weeks, WT and WA were significantly increased, which indicated that the smooth muscle cells of pulmonary arterioles in rats with pulmonary hypertension had obvious hyperplasia and hypertrophy. This could further lead to the decrease of pulmonary vascular diameter and remodeling of pulmonary arterioles. The increase of mPAP, RVHI, and resistance in pulmonary vessels confirmed that the increase of the right ventricular post load, which further resulted in the hypertrophy of the right ventricle. The above symptoms were relieved after the treatment with Ato.

**Figure 7:**
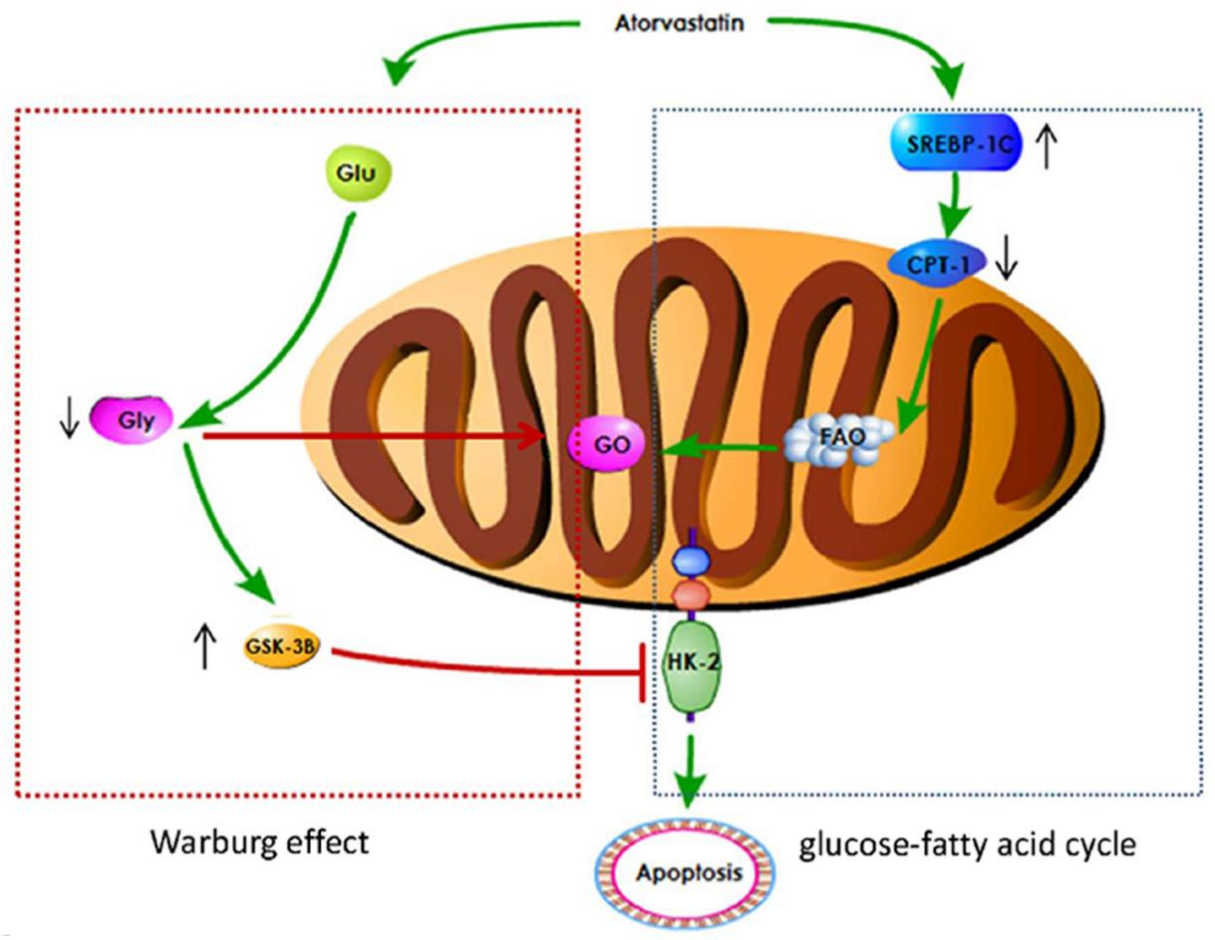
A schematic image for how Ato improve lung function of PAH rats.

Metabonomics method was used to analyze the influence of Ato and MCT on PAH animal model. Carnitine increased significantly one day after injection of MCT in Ato group, indicating that the oxidation of fatty acid β was enhanced. Meanwhile, the concentration of glucose and lipid decreased, and the concentration of glycerol increased (Tables 1, 2). Compared with Ato 1-week group, the concentration of lipid, glycerol, acetone, leucine, and isoleucine increased, but glucose decreased in the Ato 2-3 week group. Compared with the Ato 2-3 week group, amino acids, pyruvate, and acetate increased, but glucose and glycerin decreased in the Ato 4-week group (Tables 1, 2). Meanwhile, significant increase of carnitine was observed only in the Ato 1-day group indicating that the oxidation of fatty acid β was significantly inhibited by Ato. In addition, Warburg effect was not observed after the second week, suggesting that Ato significantly inhibited the oxidation of fatty acid β and promoted the aerobic oxidation of sugar. Therefore, Ato could significantly improve pulmonary vascular remodeling, which is related to the inhibition of fatty acid β oxidation and Warburg effect (Figure 7).

Abnormal expression of GSK-3β is closely related to various fibrous and vascular diseases [24, 25]. GSK-3β plays an important role in cell growth, differentiation, mutation, apoptosis and signal transduction [26]. GSK-3β could regulate the opening of mitochondrial permeability transition pore, improve mitochondrial energy production, maintain the stability of mitochondrial outer membrane, and then reduce cell apoptosis, inhibit cell hypertrophy, and promote vascular regeneration [27, 28]. During anaerobic glycolysis, hexokinase (HK-2), as a key glycolytic enzyme, is up-regulated and can shift from cytoplasm to mitochondria. HK-2 could inhibit voltage-dependent anion channels (VDACs). VDACs could transfer apoptotic mediators into the cytoplasm, thus initiating apoptosis [29, 30]. It is believed that GSK3β could promote the binding of HK-2 with VDACs inhibiting cell apoptosis [31]. In the present study, the increase level of GSK3β by Ato might exert apoptosis inhibition effect though targeting HK-2. Therefore, we speculate that Ato may be a new therapeutic target in the treatment of pulmonary hypertension by regulating GSK-3β mediated HK-2.

SREBP-1c is mainly expressed in liver tissues and adipocytes. Since the transcription regulation of adipogenesis is controlled by the quantity of SREBP-1c mRNA, SREBP-1c can regulate fat metabolism by changing its mRNA level [32, 33]. SREBP-1c was down-regulated and CPT-1 was activated in group PAH, while SREBP-1c was up-regulated and CPT-1 was inhibited in group Ato. Meanwhile, fatty acid β oxidation was increased in group PAH, but decreased in group Ato. Therefore, Ato could also inhibit vascular remodeling by regulating SREBP-1c and further affecting fatty acid β oxidation. Through KEGG database, overexpression of SREBP-1c gene can significantly affect the expression of more than 400 genes. Among them, significant positive correlation genes involved pathways include leucine and isoleucine degradation, glucose and lipid metabolism, fatty acid metabolism, polyunsaturated fatty acid biosynthesis, pyruvate metabolism, carbohydrate metabolism, bile acid biosynthesis, and lysine degradation pathways. Negative correlation genes involved pathways include adipocytokine signaling pathway. Therefore, in the MCT induced HAP model, SREBP-1c may regulate energy through multiple pathways, but the regulation of fatty acid β oxidation is the most important mechanism. Ato can inhibit fatty acid β oxidation by up-regulating SREBP-1c expression.

In conclusion, Ato can significantly improve pulmonary vascular remodeling. Ato may inhibit Warburg effect by promoting the expression of GSK-3 β and inhibiting the expression of HK-2. In addition, Ato might inhibit fatty acid β oxidation by inhibiting CPT-1 and activating SREBP-1c. Therefore, Ato inhibited PAH through regulating Warburg effect and fatty acid β oxidation.

## MATERIALS AND METHODS

### Establishment of animal model

Sprague Dawley rats (Male, 200-230 g) purchased from Shanghai Shrek laboratory animal Co., Ltd were randomly divided into three groups (10 rats/each group): Control, PAH and Ato. The rats in the group PAH were treated with MCT (60 mg/kg) through intraperitoneal injection, and the group control was treated with same amount of normal saline through intraperitoneal injection. The rats in group Ato were injected with MCT (60 mg/kg), and then treated with Ato (5 mg/kg) through gavage. Same dose of atorvastatin was given by gavage every day until the rats were sacrificed. The rats of group control and PAH were administrated with same amount of normal saline. All experiments were approved by the Ethics Committee of Fujian Medical University (2013010).

### Measurement of mPAP by right heart catheterization

The rats were anesthetized by intraperitoneal injection with ketamine (100 mg/kg) and xylazine (10 mg/kg) firstly. The right external jugular vein and the right common jugular vein were exposed. One third of the diameter of the vessel was cut obliquely at an angle of 45° C from 4 mm to proximal part of heart, and the catheter was inserted 0.5-1.0 cm into the blood vessel along the incision direction, and the catheter tip was adjusted continuously to pass smoothly. Catheter was inserted 2-3 cm further to reach the right atrium, then slowly pull and rotate the catheter to enter the right ventricle. After the appearance of typical right ventricular waveform, the pulmonary artery can be reached by entering 1 cm. When the typical pulmonary artery pressure waveform appeared, it was stabilized for 5 minutes and then the pulmonary artery systolic pressure was recorded. The experiment was performed 3 times independently.

### Determination of RVHI

After sacrificing rats, the whole heart was taken, and the left and right atria and the roots of great vessels were separated. The right ventricular free wall was cut off and water was sucked out with filter paper. The weights of right ventricular free wall (RV) and left ventricle + interventricular septum (LV + s) were weighed. Finally, RVHI = RV / (LV + s) was calculated. The experiment was performed 3 times independently.

### Image analysis of pulmonary arterioles

Six pulmonary arterioles with a diameter of 100-200 μm were randomly selected at a distance of 2 mm from the pulmonary hilum. The wall thickness (WT), external diameter (ED), total area (TA) and lumen area (LA) were measured using ipp6.0 image analysis software. According to the above data, the percentage of pulmonary artery wall thickness to diameter: WT=(2×WT/ED×100%), and the percentage of wall area (WA) to total vascular area: WA=([TA-LA]/TA×100%) were calculated. The experiment was performed 3 times independently.

### Nuclear magnetic resonance (NMR) spectrum acquisition

Serum (300 μL) collected from rats was placed on crushed ice and mixed with phosphate buffer (250 μL, 0.2 mol/L Na_2_HPO_4_, pH = 7.4). The mixed serum was centrifuged for 12000 g at 4° C for 10 min, and 500 μL supernatant was transferred to 5 mm NMR tube. Serum NMR spectra were collected at 25° C (Bruker avance III 600 NMR, Germany). The pulse sequence was cpmgpr1d (RD-90° C - (τ - 180° C - τ) n-acq) (Bruker biospin Plus Program Library), scanning times: 256, space scanning times: 16, spectrum width: 20 ppm, 2nτ = 120 ms. The fid signal was transformed into Fourier transform with 0.3 Hz window. Then, manual phase and baseline correction was performed, and calibration was performed with lactic acid (CH3, δ 1.33 ppm).

### NMR spectrum processing and pattern recognition analysis

In order to determine the information of all the metabolites in NMR spectra, the step-by-step integration of one-dimensional hydrogen spectrum was performed using mestrenova 6.1 (Mestrelab Research S.L, Spain) software. The step-by-step integration interval is divided into 0.003 ppm, and the integration area (bin) is set. The integral area of serum was 0.6-9.0 ppm. In order to eliminate the influence of water peak suppression and cross saturation of urea, the integral area of δ 4.7-6.0 ppm was defined as zero. In each spectrum, all integral values are normalized based on the total area of the spectrum. The corrected data was imported into Simca-p + 12.0 software (Umetrics AB, Sweden) for pattern recognition analysis. First, principal component analysis (PCA) was used to distinguish the metabolic patterns of different groups. The first two principal components can represent the largest information variable in the matrix. Each point on the scores plot constructed by them represents a sample. The trend of each group's aggregation on the score plot reflects the characteristics of its metabolic pattern. Orthogonal partial least squares discriminant analysis (OPLS-DA) score map was constructed by the first predictive principal component (tp) and an orthogonal component (to). The characteristic metabolites were screened by OPLS-DA model. The correlation coefficients of P = 0.05 and P = 0.01 were taken as the critical values of correlation coefficients and the variable importance in projection (VIP) values were used as the selection conditions of variables with statistical significance. The metabolites whose absolute value of correlation coefficient was greater than the critical value and VIP value was greater than 1 were identified as the metabolites contributing to the grouping. R2x (cum) and R2Y (cum) respectively indicate the percentage of variation of X variable (piecewise integral) and Y variable (grouping information) which can be explained by principal component of the model. The cumulative percentage of variation of Q2 (cum) and R2Y (cum) indicates the reliability of the model. The closer R2Y (cum) and Q2 (cum) values are to 1, the more reliable the model is.

### Real-time quantitative polymerase (RT-qPCR)

Tissues was subjected for RNA extraction with TRIzol Reagent (Solarbio, USA). RNA was added to the quantitative one step RT-PCR reaction system (Tiangen, China). RNA purity was achieved by measuring OD 260 nm/OD 280 nm using NanoDrop 8000 (Thermo). The OD 260 nm/OD 280 nm was 1.96 indicating high purity of isolated RNA. Data was analyzed using ABI 7500 Real-Time PCR System by relative quantification expression normalized to β-actin. Data were analyzed using the 2^-∆∆Ct^ method. The primers were synthesized by Sangon biosynthesis company (Shanghai China) and listed in the Supplementary Table 1. The experiment was performed 3 times independently.

### Western blotting

The proteins in tissues were lysed using lysis buffer, and same amount of samples was separated by 8% SDS-PAGE. After electrophoresis, the protein was transferred to nitrocellulose membrane (Millipore, USA). After blocking with TBST, the membranes were incubated with primary antibody (1:1000) at 4° C overnight. After washing twice with PBS, the membranes were incubated with secondary antibody (1:2000) for 2 h at room temperature. Membranes were visualized using an enhanced chemiluminescence kit (#34096, Thermo Fisher, Waltham, MA, USA) under the chemiluminescence imaging system (Bio-Rad, Hercules, CA, USA). The antibodies used in this study were listed as follows: Mouse monoclonal to GSK-3β (#ab93926, abcam, Cambridge, UK), Rabbit monoclonal to p-GSK-3β (##9323, CST, Danvers, CO, USA,), Mouse monoclonal to HK-2 (#ab209847, abcam, Cambridge, UK), Rabbit monoclonal to SREBP-1c (#ab28481, abcam, Cambridge, UK), Rabbit monoclonal to CPT-1 (#ab234111, abcam, Cambridge, UK), Mouse monoclonal to β-actin (#ab8226, abcam, Cambridge, UK), Goat anti-mouse antibody (#ab205719, abcam, Cambridge, UK), Goat anti-rabbit antibody (#ab205718, abcam, Cambridge, UK). The experiment was performed 3 times independently.

### Hematoxylin and eosin stain (H&E) staining

After deparaffinization, the tissues were stained using hematoxylin for 6 min to induce differentiation of hydrochloric acid and alcohol. Then, the tissues were stained using eosin for 20 sec. Finally, the slides were mounted and observed using microscopy (Olympus Corporation).

### Statistical analysis

PLS-DA analyses were performed on NMR data using Chenomx NMR Suite 8.0 software. The quality of the PLS-DA model was evaluated using a permutation test. Data were presented as means ± SD, and analysed by one-way analysis of variance using the Graphpad Prism 5.0 (GraphPad Software Inc., USA). P < 0.05 was considered to be significant. All experiments described above were performed at least three times.

## Supplementary Material

Supplementary Table 1

## References

[1] Coons JC, Pogue K, Kolodziej AR, Hirsch GA, George MP. Pulmonary arterial hypertension: a pharmacotherapeutic update. Curr Cardiol Rep. 2019; 21:141. 10.1007/s11886-019-1235-4 31758342

[2] Rosenzweig EB, Abman SH, Adatia I, Beghetti M, Bonnet D, Haworth S, Ivy DD, Berger RM. Paediatric pulmonary arterial hypertension: updates on definition, classification, diagnostics and management. Eur Respir J. 2019; 53:1801916. 10.1183/13993003.01916-2018 30545978 PMC6351335

[3] Bordenave J, Tu L, Savale L, Huertas A, Humbert M, Guignabert C. [New insights in the pathogenesis of pulmonary arterial hypertension]. Rev Mal Respir. 2019; 36:433–37. 10.1016/j.rmr.2019.03.003 31010759

[4] Schermuly RT, Ghofrani HA, Wilkins MR, Grimminger F. Mechanisms of disease: pulmonary arterial hypertension. Nat Rev Cardiol. 2011; 8:443–55. 10.1038/nrcardio.2011.87 21691314 PMC7097518

[5] Rafikova O, Al Ghouleh I, Rafikov R. Focus on early events: pathogenesis of pulmonary arterial hypertension development. Antioxid Redox Signal. 2019; 31:933–53. 10.1089/ars.2018.7673 31169021 PMC6765063

[6] Adhyaru BB, Jacobson TA. Safety and efficacy of statin therapy. Nat Rev Cardiol. 2018; 15:757–69. 10.1038/s41569-018-0098-5 30375494

[7] Xie L, Lin P, Xie H, Xu C. Effects of atorvastatin and losartan on monocrotaline-induced pulmonary artery remodeling in rats. Clin Exp Hypertens. 2010; 32:547–54. 10.3109/10641963.2010.503295 21091363

[8] Xu Y, Zhou S, Fang Z, Li X, Huang D, Liu Q, Zheng C. Inhibition of neointimal hyperplasia in rats treated with atorvastatin after carotid artery injury may be mainly associated with down-regulation of survivin and Fas expression. Pharm Biol. 2014; 52:1196–203. 10.3109/13880209.2014.884605 25116077

[9] Chandrasekar B, Mummidi S, Mahimainathan L, Patel DN, Bailey SR, Imam SZ, Greene WC, Valente AJ. Interleukin-18-induced human coronary artery smooth muscle cell migration is dependent on NF-kappaB- and AP-1-mediated matrix metalloproteinase-9 expression and is inhibited by atorvastatin. J Biol Chem. 2006; 281:15099–109. 10.1074/jbc.M600200200 16554298

[10] Scheller B, Schmitt A, Böhm M, Nickenig G. Atorvastatin stent coating does not reduce neointimal proliferation after coronary stenting. Z Kardiol. 2003; 92:1025–28. 10.1007/s00392-003-1022-4 14663613

[11] Hocher B, Adamski J. Metabolomics for clinical use and research in chronic kidney disease. Nat Rev Nephrol. 2017; 13:269–84. 10.1038/nrneph.2017.30 28262773

[12] Yu M, Zhu Y, Cong Q, Wu C. Metabonomics Research Progress on Liver Diseases. Can J Gastroenterol Hepatol. 2017; 2017:8467192. 10.1155/2017/8467192 28321390 PMC5339575

[13] Pallares-Méndez R, Aguilar-Salinas CA, Cruz-Bautista I, Del Bosque-Plata L. Metabolomics in diabetes, a review. Ann Med. 2016; 48:89–102. 10.3109/07853890.2015.1137630 26883715

[14] Sookoian S, Pirola CJ. Liver enzymes, metabolomics and genome-wide association studies: from systems biology to the personalized medicine. World J Gastroenterol. 2015; 21:711–25. 10.3748/wjg.v21.i3.711 25624707 PMC4299326

[15] Shao FJ, Ying YT, Tan X, Zhang QY, Liao WT. Metabonomics profiling reveals biochemical pathways associated with pulmonary arterial hypertension in broiler chickens. J Proteome Res. 2018; 17:3445–53. 10.1021/acs.jproteome.8b00316 30178671

[16] Sanders JL, Han Y, Urbina MF, Systrom DM, Waxman AB. Metabolomics of exercise pulmonary hypertension are intermediate between controls and patients with pulmonary arterial hypertension. Pulm Circ. 2019; 9:2045894019882623. 10.1177/2045894019882623 31695905 PMC6822198

[17] Zhao JH, He YY, Guo SS, Yan Y, Wang Z, Ye J, Zhang JL, Wang Y, Pang XB, Xie XM, Lin JH, Jing ZC, Han ZY. Circulating plasma metabolomic profiles differentiate rodent models of pulmonary hypertension and idiopathic pulmonary arterial hypertension patients. Am J Hypertens. 2019; 32:1109–17. 10.1093/ajh/hpz121 31350549

[18] Gomez-Arroyo JG, Farkas L, Alhussaini AA, Farkas D, Kraskauskas D, Voelkel NF, Bogaard HJ. The monocrotaline model of pulmonary hypertension in perspective. Am J Physiol Lung Cell Mol Physiol. 2012; 302:L363–69. 10.1152/ajplung.00212.2011 21964406

[19] Xiao R, Su Y, Feng T, Sun M, Liu B, Zhang J, Lu Y, Li J, Wang T, Zhu L, Hu Q. Monocrotaline induces endothelial injury and pulmonary hypertension by targeting the extracellular calcium-sensing receptor. J Am Heart Assoc. 2017; 6:e004865. 10.1161/JAHA.116.004865 28330842 PMC5533002

[20] Simonneau G, Gatzoulis MA, Adatia I, Celermajer D, Denton C, Ghofrani A, Gomez Sanchez MA, Krishna Kumar R, Landzberg M, Machado RF, Olschewski H, Robbins IM, Souza R. Updated clinical classification of pulmonary hypertension. J Am Coll Cardiol. 2013 (Suppl 25); 62:D34–41. 10.1016/j.jacc.2013.10.029 24355639

[21] Tuder RM. Pulmonary vascular remodeling in pulmonary hypertension. Cell Tissue Res. 2017; 367:643–49. 10.1007/s00441-016-2539-y 28025704 PMC5408737

[22] Şen N. [Schistosomiasis and pulmonary hypertension]. Tuberk Toraks. 2017; 65:237–44. 10.5578/tt.53798 29135402

[23] Gaine S. Pulmonary hypertension. JAMA. 2000; 284:3160–68. 10.1001/jama.284.24.3160 11135781

[24] Chilosi M, Poletti V, Zamò A, Lestani M, Montagna L, Piccoli P, Pedron S, Bertaso M, Scarpa A, Murer B, Cancellieri A, Maestro R, Semenzato G, Doglioni C. Aberrant Wnt/beta-catenin pathway activation in idiopathic pulmonary fibrosis. Am J Pathol. 2003; 162:1495–502. 10.1016/S0002-9440(10)64282-4 12707032 PMC1851206

[25] Bentley JK, Deng H, Linn MJ, Lei J, Dokshin GA, Fingar DC, Bitar KN, Henderson WR Jr, Hershenson MB. Airway smooth muscle hyperplasia and hypertrophy correlate with glycogen synthase kinase-3(beta) phosphorylation in a mouse model of asthma. Am J Physiol Lung Cell Mol Physiol. 2009; 296:L176–84. 10.1152/ajplung.90376.2008 19011050 PMC2643992

[26] Jin F, Wu Z, Hu X, Zhang J, Gao Z, Han X, Qin J, Li C, Wang Y. The PI3K/Akt/GSK-3β/ROS/eIF2B pathway promotes breast cancer growth and metastasis via suppression of NK cell cytotoxicity and tumor cell susceptibility. Cancer Biol Med. 2019; 16:38–54. 10.20892/j.issn.2095-3941.2018.0253 31119045 PMC6528454

[27] Kong J, Wang L, Ren L, Yan Y, Cheng Y, Huang Z, Shen F. Triptolide induces mitochondria-mediated apoptosis of Burkitt’s lymphoma cell via deacetylation of GSK-3β by increased SIRT3 expression. Toxicol Appl Pharmacol. 2018; 342:1–13. 10.1016/j.taap.2018.01.011 29407771

[28] Potz BA, Scrimgeour LA, Sabe SA, Clements RT, Sodha NR, Sellke FW. Glycogen synthase kinase 3β inhibition reduces mitochondrial oxidative stress in chronic myocardial ischemia. J Thorac Cardiovasc Surg. 2018; 155:2492–503. 10.1016/j.jtcvs.2017.12.127 29523407 PMC5960424

[29] Pastorino JG, Hoek JB. Regulation of hexokinase binding to VDAC. J Bioenerg Biomembr. 2008; 40:171–82. 10.1007/s10863-008-9148-8 18683036 PMC2662512

[30] Tian M, Xie Y, Meng Y, Ma W, Tong Z, Yang X, Lai S, Zhou Y, He M, Liao Z. Resveratrol protects cardiomyocytes against anoxia/reoxygenation via dephosphorylation of VDAC1 by Akt-GSK3 β pathway. Eur J Pharmacol. 2019; 843:80–87. 10.1016/j.ejphar.2018.11.016 30445019

[31] Pastorino JG, Hoek JB, Shulga N. Activation of glycogen synthase kinase 3beta disrupts the binding of hexokinase II to mitochondria by phosphorylating voltage-dependent anion channel and potentiates chemotherapy-induced cytotoxicity. Cancer Res. 2005; 65:10545–54. 10.1158/0008-5472.CAN-05-1925 16288047

[32] Knebel B, Haas J, Hartwig S, Jacob S, Köllmer C, Nitzgen U, Muller-Wieland D, Kotzka J. Liver-specific expression of transcriptionally active SREBP-1c is associated with fatty liver and increased visceral fat mass. PLoS One. 2012; 7:e31812. 10.1371/journal.pone.0031812 22363740 PMC3283692

[33] Horton JD, Shimomura I, Ikemoto S, Bashmakov Y, Hammer RE. Overexpression of sterol regulatory element-binding protein-1a in mouse adipose tissue produces adipocyte hypertrophy, increased fatty acid secretion, and fatty liver. J Biol Chem. 2003; 278:36652–60. 10.1074/jbc.M306540200 12855691

