# Supplementary material for: Influence of atorvastatin on metabolic pattern of rats with pulmonary hypertension
Source: Aging (Albany NY). 2021 Apr 22;13(8):11954–68. doi: 10.18632/aging.202898 (PMC8109122; doi:10.18632/aging.202898)
Supplement: Supplementary Table 1 [file aging-13-202898-s001.pdf]

SUPPLEMENTARY TABLE

Supplementary Table 1. Information of primers.

Primer	Sequence(5'-3')	Product length
GSK-3β	Forward: TTCTCGGTACTACAGGGCACCA<br>Reverse: GTCCTAGCAACAATTCAGCCAACA	107 bp
HK-2	Forward: GACAATGGCTGCCTGGATGA<br>Reverse: TCCCAAGTACATGCCGCTGA	114 bp
SREBP-1c	Forward: CCAGAGTAGCCCCTTGTCCTT<br>Reverse: GHATGCCCCAGCCAAACA	217 bp
CPT-1	Forward: AGGTCGGAAGCCCATGTTGTA<br>Reverse: GCTGTCATGCGCTGGAAGTC	138 bp
β-actin	Forward: CCCATCTATGAGGGTTACGC<br>Reverse: TTTAATGTCACGCACGATTTC	336 bp
